# Developmental Stage Modulates Mineral–Hormonal Adaptation After Roux-en-Y Gastric Bypass: A Translational Life-Course Study

**DOI:** 10.3390/metabo16070491

**Published:** 2026-07-13

**Authors:** Mariana Luna, Bruno Rodrigues, Andréa Ramalho

**Affiliations:** Universidade Federal do Rio de Janeiro, Rio de Janeiro 21941-590, Brazil

**Keywords:** developmental stage, mineral metabolism, metabolic adaptation, Roux-en-Y gastric bypass, parathyroid hormone, severe obesity

## Abstract

Background: Vitamin D deficiency is highly prevalent in individuals with severe obesity and may worsen after malabsorptive bariatric procedures. Adolescence represents a critical period of skeletal and metabolic maturation and may influence mineral–hormonal adaptation after Roux-en-Y gastric bypass (RYGB). Objective: To investigate whether developmental stage influences longitudinal mineral–hormonal adaptation after RYGB in adolescents and adults with severe obesity. Methods: This prospective observational cohort study included 120 participants undergoing RYGB: 60 adolescents (15–19 years) and 60 adults (20–49 years) with a history of severe obesity. Assessments were performed preoperatively and at 6 and 12 months postoperatively. Serum 25-hydroxyvitamin D, ionized calcium, phosphorus, and parathyroid hormone (PTH) were measured. Group comparisons were performed using non-parametric tests within a translational life-course framework. Results: Vitamin D inadequacy (<30 ng/mL) was highly prevalent at baseline in both groups (78.3% vs. 85.0%). Despite substantial weight loss in both cohorts, adolescents exhibited higher frequencies of hypocalcaemia and hypophosphataemia (*p* < 0.001), progressive elevation of PTH levels, and a higher prevalence of secondary hyperparathyroidism at 12 months. Adults showed a comparatively less pronounced endocrine–mineral response but also experienced persistent increases in PTH throughout follow-up. Vitamin D inadequacy remained highly prevalent in both groups despite standardized supplementation. Conclusions: Postoperative mineral–hormonal adaptation after RYGB differs according to developmental stage. Adolescents exhibited greater endocrine–mineral vulnerability during a critical period of skeletal maturation, suggesting that biological stage may influence postoperative adaptation beyond anthropometric response alone. These findings support developmental stage-specific monitoring and individualized postoperative management after metabolic surgery.

## 1. Introduction

Vitamin D (VD) plays a central role in the regulation of mineral metabolism, calcium homeostasis, skeletal remodeling, and immune function. In individuals with obesity, vitamin D deficiency (VDD) is highly prevalent and has been associated with reduced bioavailability related to adipose tissue sequestration and chronic low-grade systemic inflammation [1,2,3]. In the context of bariatric surgery, these alterations may become even more pronounced due to anatomical and physiological changes affecting nutrient absorption and metabolic regulation [4].

Roux-en-Y gastric bypass (RYGB) is one of the most effective surgical approaches for severe obesity, promoting substantial weight loss and improvement of metabolic comorbidities. However, the procedure also induces significant changes in intestinal nutrient handling and endocrine regulation, particularly involving calcium and vitamin D metabolism [4,5]. Previous studies have demonstrated that intestinal calcium absorption decreases markedly after RYGB, even in the presence of adequate VD concentrations, favoring compensatory elevation of parathyroid hormone (PTH) and increased bone turnover [5,6]. These metabolic adaptations may contribute to secondary hyperparathyroidism and long-term skeletal complications, including increased fracture risk [7].

The biological implications of these alterations may differ according to the stage of biological maturation. Adolescence represents a critical period of skeletal and metabolic maturation characterized by intense bone remodeling and peak bone mass acquisition [8,9]. Disruption of mineral homeostasis during this phase may have long-lasting consequences for skeletal health and metabolic adaptation. Previous studies have shown that adolescents undergoing bariatric surgery are particularly susceptible to nutritional and metabolic alterations after surgery [10]. Nevertheless, investigations directly comparing postoperative endocrine–mineral responses between adolescents and adults remain limited.

From a life-course perspective, biological maturation represents an important biological context that may influence the adaptive response to metabolic surgery [11]. Differences in skeletal maturation, endocrine regulation, mineral demands, and cumulative metabolic exposure contribute to distinct postoperative patterns of endocrine–mineral regulation. Exploring these differences may clarify how biological stage influences the response to the same therapeutic intervention.

In this context, the present study adopted a life-course framework to investigate whether biological maturation modulates postoperative endocrine–mineral responses after RYGB [12]. Adolescents and adults undergoing the same surgical procedure were compared to evaluate differences in the postoperative behavior of the vitamin D–calcium–PTH axis across distinct phases of skeletal and metabolic maturation. We hypothesized that postoperative endocrine-mineral responses differ according to biological context, potentially reflecting distinct patterns of metabolic plasticity and mineral vulnerability. Understanding these mechanisms could support more individualized metabolic surgery strategies and postoperative management according to biological stage.

## 2. Materials and Methods

### 2.1. Study Design and Translational Framework

This was a prospective observational cohort study with a 12-month follow-up after Roux-en-Y gastric bypass (RYGB). The study was conducted within a translational life-course framework and designed to compare postoperative endocrine–mineral adaptation between adolescents with severe obesity (G1) and adults with class III obesity (G2) undergoing the same surgical procedure.

The study design was based on the premise that developmental stage represents an important biological context that may influence adaptive responses to metabolic surgery. By examining individuals at distinct phases of skeletal and metabolic maturation, this approach integrates concepts from developmental biology, mineral metabolism, and bariatric surgery to investigate whether developmental stage is associated with differential postoperative responses of the vitamin D–calcium–phosphorus–PTH axis.

The study was specifically designed to investigate longitudinal mineral–hormonal adaptation after RYGB according to developmental stage, focusing on endocrine and mineral regulation rather than structural skeletal outcomes. Assessments were performed at three time points—preoperative (T0), 6 months (T1), and 12 months (T2) postoperatively—enabling longitudinal evaluation of metabolic alterations associated with RYGB, a procedure known to affect micronutrient absorption and mineral homeostasis [1,2].

### 2.2. Setting, Period, and Participants

Participants were recruited from a private clinic specializing in bariatric and metabolic surgery in Rio de Janeiro, Brazil.

#### 2.2.1. G1—Adolescents

Adolescents with severe obesity at baseline (T0) were included, defined as body mass index (BMI)-for-age ≥99.9th percentile. Inclusion criteria were: age between 15 years and 19 years and 11 months; sexual maturity stage IV or higher according to Tanner [3,4]; and minimum bone age of 13.5 years for females and 14.5 years for males, considering the critical period of bone mass acquisition during adolescence [5,6].

#### 2.2.2. G2—Adults

The G2 group consisted of adults aged 20 to 49 years with class III obesity (BMI ≥40 kg/m^2^) and a documented history of severe obesity since childhood and/or adolescence. This criterion was adopted to ensure that both groups shared a history of severe obesity while differing in their developmental stage at the time of surgical intervention, allowing exploration of how biological context may influence postoperative endocrine–mineral adaptation.

Only participants with complete follow-up data at T0, T1, and T2 were eligible for inclusion in the final analytical sample.

### 2.3. Ethical Aspects

All adult participants and the parents or legal guardians of adolescents provided written informed consent in accordance with national regulations. The study was approved by the Research Ethics Committee of the Clementino Fraga Filho University Hospital, Federal University of Rio de Janeiro (UFRJ), under protocol no. 011/15, and conducted in accordance with the Declaration of Helsinki.

### 2.4. Clinical Follow-Up and Supplementation

All participants underwent standardized nutritional assessment and received postoperative management according to a multidisciplinary institutional protocol. Supplementation with vitamin D (1800 IU/day) and calcium carbonate (250 mg/day) was prescribed and maintained throughout the 12-month follow-up period, consistent with international recommendations for metabolic and nutritional monitoring after bariatric surgery [7,8].

Adherence was assessed by self-report and verification of supplement packaging during follow-up visits. Adherence was considered satisfactory when at least 80% of the prescribed supplementation was consumed.

Only participants with complete anthropometric and biochemical assessments at T0, T1, and T2 and satisfactory adherence to supplementation (≥80% of the prescribed regimen) were included in the final analytical sample. Therefore, no missing data were present in the analyzed dataset.

### 2.5. Anthropometric Variables

Body weight and height were measured according to standardized procedures. BMI was calculated as weight (kg)/height^2^ (m^2^).

Waist circumference was measured at the largest abdominal sagittal diameter. For adolescents, elevated abdominal adiposity was defined according to the 90th percentile proposed by Freedman et al. [9]. For adults, internationally accepted cutoffs for abdominal obesity were applied.

### 2.6. Biochemical Variables

Blood samples (10 mL) were collected after a 12 h overnight fast at all study time points (T0, T1, and T2).

The following variables were assessed:

- 25-hydroxyvitamin D [25(OH)D], measured by high-performance liquid chromatography with ultraviolet detection (HPLC-UV) (Labtest Diagnóstica S.A., Lagoa Santa, Minas Gerais, Brazil). Cutoffs were defined as deficiency <20 ng/mL, insufficiency 20.1–29.9 ng/mL, and sufficiency ≥30 ng/mL, according to established clinical recommendations [10].
- Ionized calcium, measured by direct dosage using ion-selective electrodes and expressed in mmol/L. Hypocalcemia was defined as ionized calcium <1.20 mmol/L in adolescents and <1.00 mmol/L in adults according to age-specific reference values.
- Serum phosphorus, determined by a colorimetric method (Labtest Diagnóstica S.A., Lagoa Santa, Minas Gerais, Brazil). Hypophosphatemia was defined as serum phosphorus <4.0 mg/dL in adolescents and <2.5 mg/dL in adults according to age-specific reference ranges.
- Parathyroid hormone (PTH), measured by chemiluminescent immunoassay. Secondary hyperparathyroidism was defined as PTH >53 pg/mL based on previous studies in bariatric populations [13].

### 2.7. Sun Exposure

Baseline sun exposure (T0) was assessed using a previously validated questionnaire that recorded average daily exposure time [14]. Exposure was considered adequate when ≥20 min/day.

### 2.8. Statistical Analysis

The distribution of continuous variables was assessed using the Kolmogorov–Smirnov test and graphical inspection. Given the non-normal distribution of some biochemical variables and the sample size per group, non-parametric tests were primarily adopted.

Between-group comparisons (G1 vs. G2) at each time point (T0, T1, and T2) were performed using the Mann–Whitney U test for continuous variables and the chi-square test or Fisher’s exact test for categorical variables, as appropriate. Within-group comparisons over time (T0 vs. T1; T1 vs. T2; T0 vs. T2) were performed using the Wilcoxon signed-rank test for paired samples. Longitudinal categorical comparisons within the same group were assessed using the McNemar test when applicable. Correlations between continuous variables were evaluated using Spearman’s correlation coefficient.

A significance level of 5% (α = 0.05) was adopted. Statistical analyses were performed using SPSS software, version 21.0 (SPSS Inc., Chicago, IL, USA).

The analytical strategy was designed to characterize longitudinal endocrine–mineral adaptation after RYGB and to compare postoperative responses between developmental stages within a translational life-course framework.

## 3. Results

### 3.1. Sample Characteristics and Anthropometric Changes

A total of 120 participants were included, comprising 60 adolescents (G1) and 60 adults (G2). At baseline, both groups presented severe obesity, with similarly elevated BMI values. Baseline sun exposure was significantly higher among adolescents than adults (17.1 ± 2.0 vs. 13.2 ± 5.2 min/day, *p* < 0.001). Nevertheless, mean daily sun exposure in both groups remained below the recommended threshold, indicating insufficient preoperative sun exposure.

Adherence to postoperative supplementation was satisfactory in both groups. All participants included in the final analytical sample consumed at least 80% of the prescribed supplementation regimen throughout follow-up, consistent with the predefined eligibility criteria.

Following RYGB, both groups exhibited marked and sustained reductions in anthropometric parameters throughout follow-up (Table 1). Significant decreases in BMI, waist circumference, and waist-to-hip ratio (WHR) were observed as early as 6 months postoperatively and progressed up to 12 months. Overall weight-loss trajectories were globally similar between adolescents and adults; however, subtle between-group differences emerged over time.

Adolescents showed a trend toward greater BMI reduction at 12 months, whereas WHR reduction was significantly more pronounced in this group during follow-up (Table 2). In contrast, reductions in waist circumference were comparable between groups. These findings suggest that, despite similar overall weight loss, postoperative body composition redistribution patterns may differ according to developmental stage.

### 3.2. Vitamin D Status During Follow-Up

Serum 25-hydroxyvitamin D [25(OH)D] levels exhibited distinct longitudinal patterns between groups (Table 1). Both adolescents and adults demonstrated an initial increase at 6 months postoperatively. However, at 12 months, adolescents showed a decline toward values close to baseline, whereas adults maintained relatively higher concentrations.

From a categorical perspective, vitamin D inadequacy (<30 ng/mL) remained highly prevalent and persistent throughout follow-up in both groups, with no significant between-group differences at any time point (Table 3).

### 3.3. Biological Maturation and Endocrine–Mineral Responses

Distinct postoperative endocrine–mineral responses were observed according to biological maturation status. Ionized calcium levels remained relatively stable in adults throughout follow-up, whereas adolescents exhibited greater variability, with values returning close to baseline at 12 months (Table 1).

Categorical analysis demonstrated marked differences between groups. Hypocalcaemia was significantly more frequent among adolescents at all evaluated time points, whereas no cases were identified in adults during follow-up (Table 3). Similarly, hypophosphataemia was substantially more prevalent in adolescents across all postoperative assessments (Table 3).

In parallel, progressive elevation of PTH levels was observed in both groups over time (Table 1). Although the prevalence of secondary hyperparathyroidism increased from baseline to 12 months postoperatively in both cohorts, a more pronounced descriptive pattern was observed among adolescents, reaching 65.0% (39/60) compared with 56.7% (34/60) in adults at 12 months (Table 3).

To facilitate interpretation of the main findings, Table 4 summarizes the key anthropometric and mineral–hormonal characteristics that distinguished the two cohorts. Adolescents presented a higher baseline BMI and achieved a significantly greater absolute reduction in BMI at 12 months after surgery. Despite this more pronounced anthropometric response, adolescents exhibited lower 25(OH)D concentrations at the end of follow-up and a higher prevalence of secondary hyperparathyroidism compared with adults (Table 4).

Taken together, these findings suggest that postoperative endocrine–mineral responses after RYGB differ according to biological maturation. The coexistence of greater weight-loss magnitude and a less favorable mineral profile among adolescents supports the hypothesis that developmental factors may influence postoperative metabolic adaptation and mineral vulnerability beyond anthropometric response alone.

## 4. Discussion

The present study demonstrates that postoperative endocrine–mineral responses after RYGB differs according to biological maturation, despite substantial weight loss in both adolescents and adults. Adolescents exhibited greater postoperative mineral vulnerability, characterized by higher frequencies of hypocalcaemia, hypophosphataemia, and progressive PTH elevation throughout follow-up, whereas adults showed a pattern more compatible with chronic metabolic adaptation. Notably, adolescents presented a higher baseline BMI and achieved a significantly greater magnitude of BMI reduction at 12 months after surgery, yet they also exhibited lower 25(OH)D concentrations and a numerically higher prevalence of secondary hyperparathyroidism at the end of follow-up. Taken together, these findings suggest that biological maturation may influence postoperative mineral–hormonal regulation beyond anthropometric response alone, supporting the concept that endocrine–mineral adaptation after RYGB is shaped not only by weight loss itself but also by the biological context in which surgery occurs.

The high prevalence of vitamin D deficiency (VDD) observed in this study is consistent with the literature demonstrating reduced vitamin D bioavailability in individuals with obesity, primarily attributed to sequestration in adipose tissue and chronic systemic inflammation [1,2,3]. In the context of bariatric surgery, these alterations may become even more relevant due to changes in intestinal nutrient handling and endocrine regulation after RYGB [4]. Despite standardized postoperative supplementation, the persistence of vitamin D inadequacy throughout follow-up suggests that uniform supplementation strategies may be insufficient, reinforcing the need for individualized postoperative management, as recommended by current guidelines [13].

Alterations in mineral metabolism after bariatric surgery reflect not only reduced intestinal nutrient absorption but also the interaction with pre-existing metabolic status and biological maturity [4]. Reduced calcium absorption after RYGB has been consistently demonstrated, even in the presence of adequate serum vitamin D concentrations, favoring compensatory elevation of PTH and increased bone turnover [5,6]. In the present study, adolescents exhibited a more pronounced mineral–hormonal response characterized by progressive PTH elevation associated with persistent hypocalcaemia and hypophosphataemia throughout follow-up.

This greater postoperative vulnerability observed in adolescents may be understood in light of developmental biology and skeletal maturation. Adolescence represents a critical period of intense bone remodeling and peak bone mass acquisition [8,9]. In this context, activation of the PTH–calcium axis, although physiologically adaptive, may occur at the expense of increased skeletal mineral mobilization, potentially amplifying long-term metabolic and skeletal vulnerability [15] Previous studies have demonstrated increased bone turnover and persistent alterations in mineral metabolism during the first years after RYGB, even in patients receiving supplementation [6,13]. These findings reinforce the biological plausibility that postoperative endocrine–mineral adaptation may be particularly sensitive to the stage of skeletal and metabolic maturation.

In contrast, adults exhibited a postoperative pattern more compatible with chronic metabolic adaptation. From a developmental perspective, adults in the present study may represent a phenotype of prolonged exposure to severe obesity and chronic metabolic dysregulation, potentially contributing to distinct postoperative endocrine-mineral responses compared with adolescents undergoing earlier intervention. Previous studies have shown that secondary hyperparathyroidism may persist or even worsen after bariatric surgery, reflecting a prolonged state of mineral imbalance [15]. In addition, long-term studies have demonstrated increased fracture risk following RYGB, highlighting the clinical relevance of persistent endocrine–mineral alterations after metabolic surgery [7].

These findings should be interpreted within the broader clinical context of bariatric surgery. Although RYGB is highly effective in promoting substantial and sustained weight loss, improving metabolic health, and reducing obesity-related comorbidities [10], it may simultaneously increase skeletal vulnerability through alterations in calcium and vitamin D metabolism, secondary hyperparathyroidism, and accelerated bone remodeling [5,6,7,12,13]. Importantly, severe obesity itself is not necessarily protective for bone health, as chronic low-grade inflammation, metabolic dysfunction, and impaired bone quality have also been described in individuals with obesity [16]. Therefore, the clinical challenge is not to balance weight loss against bone health as competing outcomes, but rather to optimize both simultaneously. In this context, the greater susceptibility to mineral dysregulation observed among adolescents highlights the importance of considering skeletal maturation and mineral homeostasis when evaluating the long-term consequences of metabolic surgery [8,9,11].

Another important finding was that postoperative mineral–hormonal differences between groups were observed despite globally similar reductions in BMI and waist circumference. Although subtle differences emerged in waist-to-hip ratio trajectories, particularly among adolescents, the overall magnitude of weight loss was comparable between groups. These findings indicate that postoperative mineral adaptation cannot be explained solely by weight reduction, reinforcing the multifactorial nature of endocrine–mineral regulation after RYGB, in which developmental stage, intestinal adaptation, metabolic exposure, and skeletal demand interact.

From a clinical perspective, these findings suggest that postoperative monitoring strategies after metabolic surgery may need to consider developmental stage more carefully. Current international guidelines recommend periodic assessment of 25(OH)D, calcium, phosphorus, and PTH after bariatric surgery [7]. However, the present findings indicate that adolescents may require more individualized postoperative surveillance due to their greater endocrine–mineral susceptibility during a period of active skeletal and metabolic maturation.

These observations may also have implications for clinical decision-making in younger populations. Although the present study was not designed to compare bariatric procedures, the greater endocrine–mineral vulnerability observed among adolescents suggests that skeletal maturation and long-term mineral homeostasis should be considered alongside weight-loss efficacy when evaluating therapeutic options [8,9]. Consequently, procedure selection, supplementation strategies, and postoperative follow-up may require a more individualized approach in adolescents than in fully mature adults [11,12,17,18].

Rather than focusing primarily on structural skeletal outcomes, the present study was specifically designed to investigate endocrine–mineral adaptation to RYGB across different phases of biological maturation within a life-course framework. This approach allowed exploration of how the timing of metabolic surgery may influence postoperative mineral regulation during different phases of skeletal and metabolic maturation. Future studies integrating endocrine, mineral, and structural skeletal outcomes may further enhance understanding of these mechanisms across the life course. In addition, comparative investigations evaluating different bariatric procedures, including sleeve gastrectomy, as well as emerging incretin-based therapies, may help determine whether alternative treatment strategies can achieve comparable metabolic benefits while minimizing endocrine-mineral vulnerability, particularly during periods of active skeletal development.

**Figure 1:**
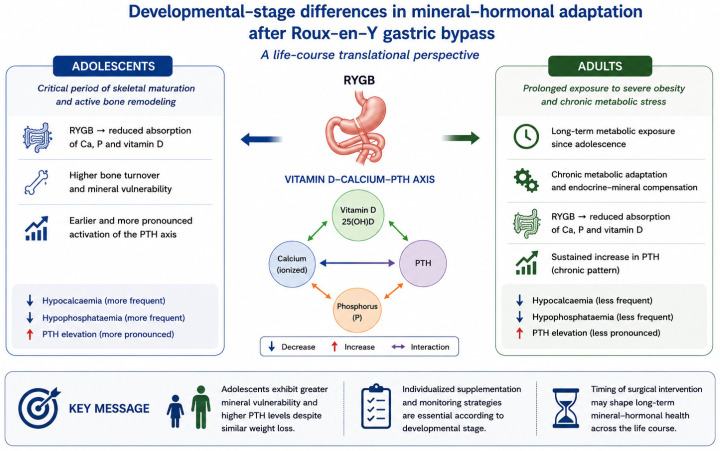
Developmental-stage differences in mineral-hormonal adaptation after Roux-en-Y gastric bypass from a translational life-course perspective. Abbreviations: RYGB, Roux-en-Y gastric bypass; 25(OH)D, 25-hydroxyvitamin D; PTH, parathyroid hormone; Ca, calcium; P, phosphorus.

Figure 1 summarizes the principal endocrine–mineral differences observed between adolescents and adults during the 12-month follow-up after RYGB.

## 5. Conclusions

This study demonstrates that postoperative endocrine–mineral responses after RYGB differ according to biological maturation. Adolescents exhibited greater postoperative mineral vulnerability, characterized by persistent hypocalcaemia, hypophosphataemia, and higher PTH levels, suggesting increased sensitivity of endocrine–mineral regulation during a critical period of skeletal and metabolic development. In contrast, adults showed a pattern more compatible with chronic metabolic adaptation and a comparatively less pronounced endocrine-mineral response.

These findings suggest that biological maturity may influence postoperative mineral regulation beyond the magnitude of weight loss alone. Within a life-course framework, the biological context in which metabolic surgery occurs may contribute to distinct patterns of endocrine–mineral regulation and metabolic vulnerability. Accordingly, postoperative management after RYGB should incorporate age- and maturity-sensitive monitoring and individualized supplementation strategies to optimize long-term metabolic and skeletal health.

## Figures and Tables

**Table 1:** Anthropometric and biochemical variables over follow-up in adolescents (G1) and adults (G2) undergoing Roux-en-Y gastric bypass.

Variable	Time	Adolescents (G1)	Adults (G2)	*p* (T0–T1)	*p* (T1–T2)	*p* (T0–T2)
BMI (kg/m^2^)	T0	47.10 ± 8.35	43.64 ± 4.96	<0.001	<0.001	<0.001
	T1	33.06 ± 7.16	31.45 ± 4.01			
	T2	28.61 ± 7.73	28.62 ± 4.07			
Waist circumference (cm)	T0	125.23 ± 16.86	122.19 ± 15.09	<0.001	<0.001	<0.001
	T1	96.92 ± 14.65	91.82 ± 11.04			
	T2	88.58 ± 13.70	86.42 ± 11.26			
Waist-to-hip ratio (WHR)	T0	1.11 ± 0.12	0.94 ± 0.07	<0.001	0.010/0.037	<0.001
	T1	0.97 ± 0.16	0.85 ± 0.07			
	T2	0.91 ± 0.15	0.84 ± 0.07			
25(OH)D (ng/mL)	T0	22.78 ± 8.27	21.13 ± 7.50	0.010/<0.001	<0.001/0.421	0.332/0.006
	T1	25.05 ± 8.26	25.76 ± 7.41			
	T2	21.36 ± 7.47	25.01 ± 10.73			
Ionized calcium (mmol/L)	T0	1.12 ± 0.15	1.22 ± 0.33	<0.001	<0.001	0.590/0.876
	T1	1.29 ± 0.34	1.34 ± 0.34			
	T2	1.11 ± 0.12	1.21 ± 0.33			
Phosphorus (mg/dL)	T0	3.35 ± 0.63	3.27 ± 0.61	0.070/0.491	0.421/0.157	0.023/0.569
	T1	3.50 ± 0.56	3.33 ± 0.58			
	T2	3.56 ± 0.57	3.22 ± 0.50			
PTH (pg/mL)	T0	47.97 ± 25.62	45.26 ± 25.36	0.055/0.321	0.028/0.018	<0.001
	T1	56.04 ± 21.81	49.27 ± 21.56			
	T2	63.82 ± 22.43	56.21 ± 24.69			

Legend: BMI: body mass index; WHR: waist-to-hip ratio; 25(OH)D: 25-hydroxyvitamin D; PTH: parathyroid hormone. T0: preoperative; T1: 6 months postoperative; T2: 12 months postoperative. Continuous variables are expressed as mean ± standard deviation (SD). Within-group comparisons (T0–T1, T1–T2, and T0–T2) were performed using the Wilcoxon signed-rank test for paired samples.

**Table 2:** Absolute anthropometric changes (Δ) from baseline following Roux-en-Y gastric bypass in adolescents (G1) and adults (G2), with between-group comparisons.

Variable	Adolescents (G1) Δ T0–T1	Adults (G2) Δ T0–T1	*p*-Value	Adolescents (G1) Δ T0–T2	Adults (G2) Δ T0–T2	*p*-Value
BMI (kg/m^2^)	−14.04 ± 6.42	−12.18 ± 3.91	0.06	−18.49 ± 7.10	−15.01 ± 4.55	0.002
WC (cm)	−28.31 ± 17.94	−30.38 ± 9.84	0.44	−36.65 ± 16.49	−35.77 ± 10.91	0.73
WHR	−0.145 ± 0.146	−0.090 ± 0.064	0.008	−0.193 ± 0.141	−0.098 ± 0.069	<0.001

Legend: BMI: body mass index; WC: waist circumference; WHR: waist-to-hip ratio. T0: preoperative; T1: 6 months postoperative; T2: 12 months postoperative. Continuous variables are expressed as mean ± standard deviation (SD). Δ represents absolute change from baseline (T0). Between-group comparisons (G1 vs. G2) were performed using the Mann–Whitney U test.

**Table 3:** Prevalence of inadequacy over time (T0, T1, T2) in adolescents (G1) and adults (G2).

Marker (Study Cutoff)	Time	G1 n/N (%)	G2 n/N (%)	*p*-Value (Between Groups)
Vitamin D < 30 ng/mL	T0	47/60 (78.3)	51/60 (85.0)	1.000
	T1	45/60 (75.0)	50/60 (83.3)	0.825
	T2	47/60 (78.3)	53/60 (88.3)	0.221
Hypocalcemia (group-specific cutoff)	T0	33/60 (55.0)	0/60 (0.0)	<0.001
	T1	14/60 (23.3)	0/60 (0.0)	<0.001
	T2	38/60 (63.3)	0/60 (0.0)	<0.001
Hypophosphatemia (group-specific cutoff)	T0	48/60 (80.0)	2/60 (3.3)	<0.001
	T1	40/60 (66.7)	4/60 (6.7)	<0.001
	T2	46/60 (76.7)	6/60 (10.0)	<0.001
PTH > 53 pg/mL	T0	20/60 (33.3)	21/60 (35.0)	1.000
	T1	26/60 (43.3)	21/60 (35.0)	0.195
	T2	39/60 (65.0)	34/60 (56.7)	0.131

Legend: PTH: parathyroid hormone. T0: preoperative; T1: 6 months postoperative; T2: 12 months postoperative. Values are expressed as absolute frequency (n/N) and percentage (%). Between-group comparisons were performed using the chi-square test or Fisher’s exact test, as appropriate. Group-specific cutoffs were applied for calcium and phosphorus according to age-adjusted reference values.

**Table 4:** Summary of key anthropometric and mineral–hormonal characteristics distinguishing adolescents and adults during the 12-month follow-up after Roux-en-Y gastric bypass.

Variable	Adolescents (*n* = 60)	Adults (*n* = 60)	*p*-Value
Baseline BMI (kg/m^2^)	47.10 ± 8.35	43.64 ± 4.96	<0.001
Absolute BMI reduction (Δ T0–T2, kg/m^2^)	−18.49 ± 7.10	−15.01 ± 4.55	0.002
25(OH)D at 12 months (ng/mL)	21.36 ± 7.47	25.01 ± 10.73	0.006
Secondary hyperparathyroidism at 12 months [n (%)] *	39/60 (65.0)	34/60 (56.7)	0.131

* Defined as PTH > 53 pg/mL. Values are expressed as mean ± standard deviation or absolute frequency (n) and percentage (%), as appropriate. BMI: body mass index; 25(OH)D: 25-hydroxyvitamin D; PTH: parathyroid hormone; T0: preoperative; T2: 12 months postoperative.

## Data Availability

The raw data supporting the conclusions of this article will be made available by the authors on request.
